# Targeting neuro-immune systems to achieve cardiac tissue repair following myocardial infarction: A review of therapeutic approaches from in-vivo preclinical to clinical studies

**DOI:** 10.1016/j.pharmthera.2023.108397

**Published:** 2023-03-28

**Authors:** Sarah Smith, Raimondo Ascione

**Affiliations:** Bristol Heart Institute and Translational Biomedical Research Centre, Faculty of Health Science, University of Bristol, Bristol, UK

**Keywords:** Myocardial infarction, Cardiac repair, Ischemic heart failure, Neuro-immune response, Therapeutic approaches

## Abstract

Myocardial healing following myocardial infarction (MI) toward either functional tissue repair or excessive scarring/heart failure, may depend on a complex interplay between nervous and immune system responses, myocardial ischemia/reperfusion injury factors, as well as genetic and epidemiological factors. Hence, enhancing cardiac repair post MI may require a more patient-specific approach targeting this complex interplay and not just the heart, bearing in mind that the dysregulation or modulation of just one of these systems or some of their mechanisms may determine the outcome either toward functional repair or toward heart failure.

In this review we have elected to focus on existing preclinical and clinical in-vivo studies aimed at testing novel therapeutic approaches targeting the nervous and immune systems to trigger myocardial healing toward functional tissue repair. To this end, we have only selected clinical and preclinical in-vivo studies reporting on novel treatments targeting neuro-immune systems to ultimately treat MI. Next, we have grouped and reported treatments under each neuro-immune system. Finally, for each treatment we have assessed and reported the results of each clinical/preclinical study and then discussed their results collectively. This structured approach has been followed for each treatment discussed. To keep this review focused, we have deliberately omitted to cover other important and related research areas such as myocardial ischemia/reperfusion injury, cell and gene therapies as well as any ex-vivo and in-vitro studies.

The review indicates that some of the treatments targeting the neuro-immune/inflammatory systems appear to induce beneficial effects remotely on the healing heart post MI, warranting further validation. These remote effects on the heart also indicates the presence of an overarching synergic response occurring across the nervous and immune systems in response to acute MI, which appear to influence cardiac tissue repair in different ways depending on age and timing of treatment delivery following MI. The cumulative evidence arising from this review allows also to make informed considerations on safe as opposed to detrimental treatments, and within the safe treatments to ascertain those associated with conflicting or supporting preclinical data, and those warranting further validation.

## 1 Introduction

Myocardial infarction (MI) activates an intricate series of multiorgan biological, inflammatory, reparative/healing or scarring processes. The type, intensity and synergy of these integrated responses post MI may influence the cardiac tissue healing either toward functional repair or toward scarring/heart failure (Frangogiannis, 2012; Nahrendorf, Pittet, & Swirski, 2010; Scalise et al., 2021). Soon after MI, an intense early sterile inflammatory response occurs promoting recruitment of activated immune cells from the circulation, bone marrow and from within the heart which enables the digestion/phagocytosis and removal of necrotic cells and extracellular matrix debris. This process is collectively referred to as the early inflammatory phase of cardiac repair. The subsequent reparative phase includes the mediation of inflammatory responses, fibroblast and myofibroblast activation/proliferation, neoangiogenesis and scar formation (Frangogiannis, 2012; Nahrendorf et al., 2010). In humans, it takes approximately 2 months for mature scar to form from the initial acute MI event (Scalise et al., 2021).

Myocardial scarring, as a healing process, may occur because the adult heart has minimal functional reparative capabilities to compensate for the level of cardiomyocytes loss and impaired cardiac function caused by the ischemia/reperfusion injury (Zlatanova, Pinto, & Silvestre, 2016). Historic conjecture was that adult cardiomyocytes were unable to regenerate, but this has now been rescinded. A number of key studies have reported proliferation and renewal of human cardiomyocytes (Bergmann et al., 2009; Kajstura et al., 2010; Mollova et al., 2013). However, the annual turnover rate is a mere 1%, which is also subject to fluctuation depending on the condition and age of the heart, genetic phenotype and patient risk profile. Since it is estimated that up to 1 billion cardiomyocytes are destroyed as a result of an MI (Sahara, Santoro, & Chien, 2015), it is obvious that in the adult heart the very limited rate of cardiomyocyte turnover is totally eclipsed by the large rate of necrosis. Hence, a large amount of extracellular matrix deposition occurs as a protective wound healing mechanism to prevent cardiac rupture, albeit ata significant cost to cardiac function.

Aberrations in the early inflammatory phase response may impact on the late resolution phase which can lead to extreme pathological remodelling of the left ventricle and enhanced heart failure (Prabhu & Frangogiannis, 2016). It is therefore possible that the overall effect, either detrimental or beneficial, of late resolution phase of myocardial injury on the cardiac tissue repair may be influenced by multiple and integrated molecular and cellular processes which are critically linked to the activity and timing of immune responses.

**Fig. 1:**
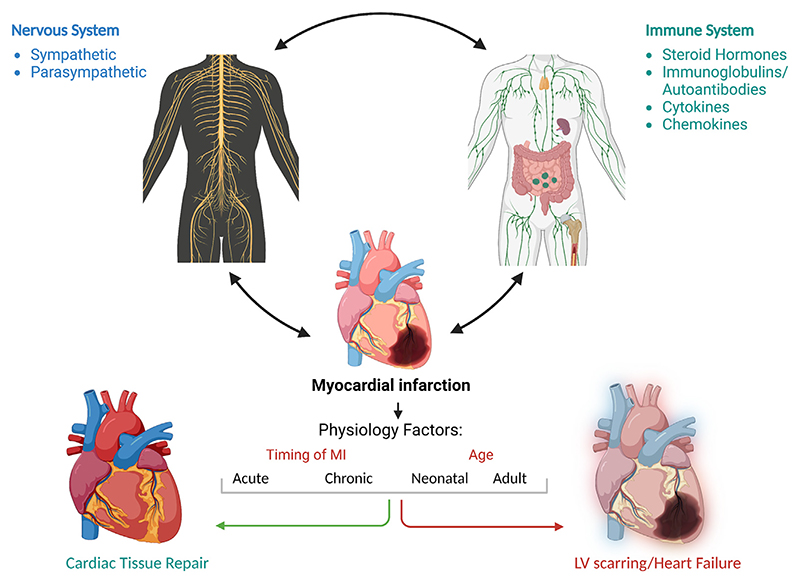
Schematic of interplay between immune and nervous systems and cardiac tissue repair following MI.

The progression of myocardial healing after MI toward either functional tissue repair or excessive scarring/heart failure may depend on the diverse and plastic interplay of the neuro-immune and inflammatory systems response, and how these impacts the integrated processes of myocardial tissue preservation, cellular proliferation/recruitment and/or differentiation as well as tolerance to ischemia/reperfusion injury (Uygur & Lee, 2016) (Fig. 1; created by the authors at http://Biorender.com). Key factors triggering functional cardiac repair as opposed to excessive scarring may include the preservation of the myocardium to limit tissue damage and cardiac dysregulation, cardiomyocyte survival to limit the extent of fibrosis and scarred territory, reduced pro-inflammatory responses to modulate the tissue repair, cellular crosstalk to promote survival and initiation of adaptive immune responses, tissue neoangiogenesis to maintain tissue architecture and blood supply to save cardiomyocytes at risk, proliferation and cell cycle activation of mitotic cardiomyocytes, and other factors such as ageing, genetic phenotype, patient risk profile. The effectiveness of cardiac tissue repair may be influenced by the synergic interplay of all these aspects, whilst it is also plausible that the dysregulation of just one of these factors might be detrimental (Broughton et al., 2018a).

The aim of this review was to evaluate and integrate the existing evidence arising from preclinical and clinical in-vivo studies aimed at testing new therapeutic approaches targeting the nervous and/or the immune systems to trigger myocardial tissue repair post MI. To keep this focus, we have deliberately omitted to include other relevant research areas such as myocardial ischemia/reperfusion injury and attempts to myocardial regeneration via cell and gene therapies. However, there are already a number of comprehensive reviews on recent advances of cell therapy as methods of immunoregulation (Farache Trajano & Smart, 2021; Murata, Ikegawa, Minatoya, & Masumoto, 2020; Ghiroldi et al., 2018; Gyongyosi, Wojakowski, Navarese, Moye, & Investigators, 2016; Mazzola and Di Pasquale, 2020).

## 2 Methods

We searched the literature with a focus only on clinical and preclinical in-vivo studies reporting on molecules or drugs used to target the neuro-immune systems to improve cardiac tissue repair and cardiac function following MI. All in-vivo preclinical studies included in this review and grouped by treatment type used i.e. molecules or drugs are shown in Tables 1 & 2 respectively. All clinical studies are described in text for each treatment evaluated. Next, we grouped and reported the selected treatments by each neuro-immune system area targeted including nervous system, immune suppression (steroids, immunoglobulins and auto-antibodies), non-steroidal anti-inflammatories, cytokine therapies (IL-1, IL-6, IL-11, TNF-α), and chemokine therapies (CCL2, CCL5, CCL25, CXCL12). Then, for each treatment, we assessed and reported the results of each clinical/preclinical study and then discussed the results of those studies collectively within each neuro-immune system area. To keep this review focused neuro-immune systems and cardiac repair, we deliberately omitted to cover other important and related research areas such as myocardial ischemia/reperfusion injury and myocardial regeneration via cell and gene therapies.

### 2.1 Nervous system

The autonomic nervous system regulates many functions of the human body. Since the first pioneering work a century ago, demonstrating that denervation inhibits limb regeneration in newts (Stocum, 2011), consistent reporting support that nerves contribute to regeneration in various tissues in vertebrates and invertebrates (Kumar & Brockes, 2012). Indeed, the mammalian heart is innervated by both sympathetic and parasympathetic nerves. Tada coined the phrase ‘super systems’ to describe both the nervous system and immune system as integrated processes which are entwined to maintain body homeostasis (Tada, 1997). As matter of fact, it has been demonstrated that autonomic nerves play an important role in sensing/controlling inflammation and even modulating immune responses (Kenney & Ganta, 2014). Both adrenergic and cholinergic receptors are expressed on a variety of resident and infiltrating cardiac immune cells (Lu & Wu, 2021; Tanner, Maitz, & Grisanti, 2021) whilst adverse cardiac remodelling leads to maladaptive neurohormonal activation (Fernandez & Canty, 2015). In a murine MI model, 3D imaging of the infarcted heart revealed that sympathetic nerves penetrate into the myocardium and follow an aligning path with coronary vessels but are also dispersed independently around the coronary vasculature. Furthermore, denervation is observed at the infarct site whilst hyperinnervation (nerve sprouting) dominates the border zone territory (Yokoyama et al., 2017). In addition, parasympathetic nerves have been shown to innervate the atria at discrete locations, run along the axis of major coronary vessels and to be present in ventricular walls (Ardell & Randall, 1986; Randall, Milosavljevic, Wurster, Geis, & Ardell, 1986; Singh et al., 1996). This intriguing triangulation between nervous system, immune system and cardiac tissue repair or excessive scarring following MI is extremely interesting, and more needs to be done in this area to better understand the overarching mechanisms and signalling processes involved across these systems and how these could be better harnessed to benefit patients.

#### 2.1.1 Neonatal cardiac repair/regeneration

The neonate mammalian heart is known to regenerate (Lam & Sadek, 2018). In the first week of life neonatal mice can fully regenerate their hearts, although this capacity diminishes shortly after this time (Porrello et al., 2011). Similar early cardiac regenerative capabilities have also been observed in human neonates experiencing a MI immediately after birth (Haubner et al., 2016). Accordingly, 1–2 days old pigs show signs of obvious cardiac regeneration after a MI with restoration of contractile function, cardiomyocyte replenishment and minimal fibrosis, although this reparative capability is lost in older neonatal and adult pigs (Ye et al., 2018; Zhu et al., 2018). The key role of the nervous system in neonatal cardiac regeneration was confirmed in a study demonstrating that the subepicardial sympathetic nerves in neonate mice reinnervate the ventricular myocardium following induction of apical resection injury, whereas the hearts of sympathectomized mice exhibited extensive collagenous scar formation, heart failure and no evidence of regeneration. Similarly, the denervation of sub-epicardial nerves using a chemical inhibitor in a neonate murine heart led to myocardial injury and fibrosis (White, Gordon, Balkan, & Hare, 2015). The role of the nervous system is also confirmed by a study in mouse neonates where disruption of parasympathetic/cholinergic signalling diminished cardiomyocyte cell cycle activity following injury with transcriptional profiling indicating a dysregulation in the inflammatory and immune response following injury (Mahmoud et al., 2015).

#### 2.1.2 Adult cardiac repair/healing

Preclinical studies have assessed whether nervous bioelectrical stimulation improve the reparative capacity of the injured adult heart. Sympathetic nerve stimulation applied for 10 weeks following MI in pig led to improved LVEF and remodelling versus controls, with histology showing diffuse sympathetic nerve sprouting/reinnervation in the infarct and peri-infarct regions (Liao et al., 2015). This is an interesting finding indicating that early sympathetic nerve stimulation after MI is effective, although mechanistic insights behind these beneficial effects are not clear. In another study focusing on a canine model of post-ischemic heart failure (HF), the parasympathetic stimulation of the vagal nerve over three months also improved LVEF and inflammatory biomarkers (Hamann et al., 2013). Hence, together these studies appear to suggest that acute and chronic MI could be treated separately or consecutively through early sympathetic nerve stimulation in the acute phase MI and parasympathetic stimulation in the chronic phase MI. Accordingly, vagal nerve stimulation has also been associated with a cardio-protective effect in ischemia-reperfusion models, leading to smaller infarct size following prolonged stimulation (Katare et al., 2009; Shinlapawittayatorn et al., 2013). Similar results have been achieved also using pharmacological therapies. Pyridostigmine (Pyr) was used to positively modulate vagal nerve activity in a rat model of MI leading to an increase in anti-inflammatory type 2 macrophages (M2) in the infarct and peri-infarct areas as well as an increase in circulating immune suppressing Treg cells (Rocha et al., 2016). In addition, rats treated with Pyr for 60 days prior to an MI showed a lower heart rate, improved LVEF, and reduced LV expression of inflammatory markers (IFN-γ, IL-6, and IL-1β) compared to controls (Barboza et al., 2019).

### 2.2 Immune suppression

A plethora of research has elucidated many timely and distinct events of the immune system involved in cardiac repair/healing (reviewed in Broughton et al., 2018b; Epelman, Liu, & Mann, 2015; Zlatanova et al., 2016). The administration of broad-spectrum immuno-suppressants in post-MI patients has produced both promising and conflicting results, with systematic review of clinical trials highlighting studies reporting negative effects in post-infarct healing. Examples of broad spectrum immuno-suppressants used in those trials included steroid hormones, anti-inflammatory drugs, or intravenous immunoglobulin (IVIg) (Panahi et al., 2018). A number of studies have also investigated the impact of modulation of specific immune pathways targeted by broad immune-suppressant drugs used in clinical practice.

#### 2.2.1 Steroid hormones

Endogenous glucocorticoids suppress cell mediated immune responses through binding to glucocorticoid receptors and activating transcription factor pathways such as NF-KB, to up-regulate the expression of anti-inflammatory genes and represses proinflammatory genes affecting activation, proliferation and apoptotic processes of immune cells (Banuelos et al., 2016; Leung & Bloom, 2003; Rhen & Cidlowski, 2005). In mammals, circulating glucocorticoid levels rise dramatically shortly before birth as a normal process to promote maturation of fetal organs and to prepare the fetus for the physiological transition outside of the uterus (Bird, McDougall, Seow, Hooper, & Cole, 2015; Fowden, Li, & Forhead, 1998). Hence, synthetic glucocorticoids are used for women at risk of delivering premature babies which reduces the incidence and severity of respiratory distress and mortality in new-born offspring. This treatment mimics the increase of endogenous levels which normally occur shortly before birth and therefore prematurely activates the maturational events reminiscence in full term, including improving cardiovascular function (Jellyman, Fletcher, Fowden, & Giussani, 2020; Kim et al., 2014; Liggins & Howie, 1972). Glucocorticoid receptors (GR) are ubiquitously expressed on all nucleated cells having a wide and varied effect on different cell types and associated inflammatory responses (Cain & Cidlowski, 2017).

Maturation events exit cardiomyocytes from the cell cycle reducing their proliferative effects after birth (Ahuja, Sdek & MacLellan, 2007). A mouse model with cardiomyocyte-specific deletion of the glucocorticoid receptor (cGR-KO) showed deterioration from 3 months of age, including a decline in LV ejection fraction, increased LV mass and cardiac hypertrophy, and premature death (Oakley et al., 2013). This suggests glucocorticoids playing a key role in switching the cardiomyocyte phenotype from exiting the cell cycle to one of hypertrophy, a switch subsequently confirmed during early post-natal development in the same model. Also, in a MI model using cGR-KO mice, cardiomyocytes were more proliferative 10 days post-MI in the border zone region, suggesting tissue regeneration, with reduced scar formation. However, whilst ablation of GR demonstrates enhanced cardiac tissue repair following MI, longer term prognosis was poor (Pianca et al., 2022).

Additional complexity is due to the high affinity of GC for binding to the mineralocorticoid receptor (MR). In contrast to GR, MR has a localised expression and is found in non-epithelial tissue of the heart (Oakley & Cidlowski, 2015). The Eplerenone Post–Acute Myocardial Infarction Heart Failure Efficacy and Survival Study (EPHESUS) demonstrated that eplerenone (a selective MR inhibitor), used in conjunction with standard therapy in patients with HF post-MI, reduced morbidity and mortality in these patients (Greenberg, Zannad, & Pitt, 2006; Pitt et al., 2003). Accordingly, a study in a transgenic MI mouse model using cardiomyocyte-specific deletion of MR found a reduction in adverse cardiac remodelling associated with the suppression of NF-kB leading to reduced apoptosis early following MI (Fraccarollo et al., 2011).

Another study compared the reduced lifespan in transgenic mice with cGR-KO to those with cMR-KO, along with a double receptor knock out, cGR-cMR-KO. Both the cMR-KO and the double knock out animals displayed normal lifespans compared to cGR-KO. Furthermore, cGR-KO hearts exhibited adverse LV remodelling and raised inflammatory markers, whereas the cMR-KO hearts had normal morphology and structure. cGR-cMR-KO hearts were resistant to the adverse LV remodelling observed in cGR-KO hearts (Oakley et al., 2019). These findings suggest that GR ablation does not prevent premature death and LV remodelling when MR and its associated signalling pathway remain intact. By contrast, MR ablation is not associated with detrimental effects while eliminating both receptors, i.e. all forms of glucocorticoid signalling in cardiomyocytes, is less detrimental compared to GR ablation alone. Collectively, these findings suggest that selective activation of GR and selective inhibition of MR may represent a novel therapeutic approach for cardiac regeneration, although further preclinical validation of these preliminary findings in mice is needed possibly in large animals.

Another approach to modulate inflammation following cardiac injury might be harnessing endogenous GC levels. These are physiologically elevated in response to an ischemic insult through the activation of the hypothalamic–pituitary–adrenal axis (Morrison et al., 1976) or by regeneration within cells from inert metabolite such as cortisone and 11-dehydrocorticosterone 11-DHC. GC is regenerated by the intracellular enzyme 11β-hydroxysteroid dehydrogenase type 1 (11β-HSD1), with evidence that GC levels can be modulated by IL-1 (Tomlinson et al., 2001) and GC (Chapman et al., 2006), leading to a localised amplification of GC levels during inflammation (Chapman, Holmes, & Seckl, 2013; Sun & Myatt, 2003). Accordingly, a mouse MI model of globally 11β-HSD1-deficient mice showed improved LVEF and reduced infarct size and LV remodelling at 8 weeks post-MI (White et al., 2016), while in a mouse model of non-perfused cardiac remodelling, an inhibitor of 11β-HSD1 reversed established LV hypertrophy and dysfunction (Gordon et al., 2014). This work has led to the development of pharmacological inhibitors of 11β-HSD1 (Anderson & Walker, 2013; Sooy et al., 2015) with clinical trial data in diabetic patients showing improved metabolic outcomes (Anderson & Walker, 2013). This approach could pave for the development a similar therapeutic approach for cardiac tissue repair post MI.

#### 2.2.2 Immunoglobulins and auto-antibodies

IVIg, a blood product derived from the serum of between 1000 and 15,000 donors per preparation, is an effective treatment for a variety of disorders and diseases with basis in neurology, immunology, dermatology, nephrology, rheumatology and ophthalmology (Jolles, Sewell, & Misbah, 2005). Immunoglobulins are glycoprotein molecules produced by plasma cells acting as an integral part of the immune response, being able to recognize and bind to exogenous antigens such as bacteria or viruses to trigger their removal (Liu, Cao, & Li, 2020). In a clinical trial IVIg were used to treat patients with chronic HF. They produced an obvious anti-inflammatory effect, measured by IL-1R, TNF and IL-10, leading to a significant improvement in LVEF (Gullestad et al., 2001), while another trial found no benefit associated with the use of IVIg in patients with dilated cardiomyopathy (DCM) (McNamara et al., 2001). These findings may warrant further probing to ascertain if IVIg might trigger cardiac repair, bearing in mind that IVIg effectiveness may be influenced by dose, immunoglobulin type and type of cardiac disease (Jolles et al., 2005). Preliminary studies have assessed the combined treatment of immunoadsorption-IVIg by administering to patients first a treatment for the non-specific immunoadsorption to reduce autoantibody levels, followed by IVIg treatment, showing improved myocardial function in patients with DCM and reduced myocardial inflammation (Felix et al., 2000; Staudt et al., 2001). These findings have led to a large randomized trial in 200 DCM patients assessing the efficacy of immunoadsorption-IVIg with an estimated completion date of June 2022 (https://clinicaltrials.gov/ct2/show/NCT00558584).

Cardiac autoantibodies commonly detected are those targeting antigens of myosin, troponin I, β1-AR, anti-Na-K-ATPase, anti-endothelin A receptor, muscarinic receptors, and anti-AT-1 receptor. Whether these autoantibodies are disease-modifying agents or solely biomarkers of cardiac injury and HF is still uncertain (Keppner et al., 2018). It has been suggested that β1-autoantibodies play a role in causation or progression of HF, although β1-AR antibodies are present in patients with HF but also in healthy individuals (Dungen et al., 2020). β1-AR autoantibodies have been associated with a pathological effect through activation of β1-adrenergic receptors thereby prolonging their active conformation. The β1 adrenergic receptor is a G-protein-coupled receptor and its activation initiates a cAMP-dependent pathway through adenylyl cyclase (Alhayek & Preuss, 2021). Of note, circulating autoantibodies against β1-AR have been observed in 30–40% of DCM HF patients (Iwata et al., 2001; Jahns et al., 1999) with suggestion that they promote myocyte apoptosis (Staudt et al., 2003), sustained calcium influx resulting in electrical instability of the heart (Christ et al., 2001) and contractile dysfunction (Fu et al., 2014). Clinically, β-blockers are used to inhibit these adverse cardiac effects and whilst an effective management strategy they also generate a feedback mechanism resulting in the upregulation of AR receptors. This has been suggested as a potential mechanism through which cardiac autoimmunity may be maintained. Hence, new therapies to block or modulate AR receptor activities are being developed as a cost-effective alternative to immunoadsorption. Intravenous neutralization of autoantibodies using small soluble molecules, which specifically target and deplete β_1_-adrenergic autoantibody levels against the β_1_-adrenergic receptor, have so far demonstrated that aptamers can successfully neutralize G-protein-coupled autoantibodies in vivo in humans but its wider application is still in its infancy (Dungen et al., 2020).

### 2.3 Non-steroidal anti-inflammatories

Cyclooxygenase (COX) is an inducible enzyme that drives inflammation and is the therapeutic target of nonsteroidal anti-inflammatory drugs (NSAIDs), including aspirin. There are two major isoforms of COX; COX1 and COX2, and both mediate prostaglandin synthesis (Warner, Nylander, & Whatling, 2011). Aspirin acts through non-competitive, irreversible acetylation of COX. Nucleated cells synthesize COX-1 and COX-2, which recovers prostaglandin synthesis even in the presence of aspirin. In contrast, the effects of aspirin on COX in platelets is irreversible. In platelets, aspirin effects on COX-1 prevents production of thromboxane A2 and as such has an irreversible effect during the life span of the cell resulting in sustained inhibition of platelet aggregation, thus mediating aspirins cardioprotective effects (Huang & Frangogiannis, 2018). However, level of response to aspirin, in terms of platelets inhibition, are patient-specific indicating that other factors are involved. Aside from aspirin, NSAID competitively and reversibly inhibits COX (Vonkeman & van de Laar, 2010). The ratio of expression of COX isoforms are cell type dependent (Warner et al., 2011). COX-2 selective NSAIDs were developed to alleviate anti-platelet effects of traditional NSAIDs via COX-1 inhibition (Sudano et al., 2012) and to avoid gastrointestinal side effects observed with earlier anti-inflammatory drugs (Boulakh & Gislason, 2016).

In the infarcted heart, COX-2 activity is triggered by acute inflammation, ischaemia and stress (Huang & Frangogiannis, 2018). Parecoxib, a selective COX-2 inhibitor, has been associated with prevention/reduction of adverse LV remodelling and cardiomyocyte death and improved arteriolar density in small rodent MI models (Abbate et al., 2006; Straino et al., 2007). Similar studies in mice revealed that Parecoxib reduces mortality and apoptosis in non-reperfused MI, with no beneficial effects detected in reperfused MI (Salloum et al., 2009). However, the use of valdecoxib and its prodrug parecoxib in a trial with 1671 patients to control postoperative pain following coronary-artery bypass grafting was associated with a higher rate of MI, cardiac arrest, stroke, and pulmonary embolism compared to placebo controls (Nussmeier et al., 2005). Hence, there is a general perception that broad based NSAID immune suppression may trigger adverse cardiovascular events (Panahi et al., 2018) even if preclinical results in mice are promising. Similarly, while a meta-analysis in patients suggested no increased risk of hypertension, stroke or MI associated with the use of the COX-2 selective NSAID, celecoxib (Chan et al., 2009), the use of the same drug in a porcine MI model using celecoxib at 400 mg twice daily for 6 weeks was associated with increased mortality and enhanced adverse LV remodelling. The authors highlighted that the study began administration of celecoxib following the MI and it remains unknown whether treatment with the drug prior to MI or during the chronic MI phase or at a lower dose or for a shortened time period might have led to different outcome. Additionally, lower doses did not sufficiently inhibit COX-2 activity in pigs (Timmers et al., 2007) raising the question as to whether partial inhibition of COX-2 activity may produce a beneficial response. A recent study using a cryoinjury neonate mouse model has suggested therapeutic efficacy when celecoxib is administered on days 0, 1 and 2 only following injury, with reduced cardiac hypertrophy, fibrosis, inhibition of cytokine expression, increased recruitment of M1-like macrophages and improved cardiac function at 4 weeks (Zhao et al., 2021). The obvious differences between mice and pig models used might explain the different responses observed in the preclinical studies described above.

Another COX-2 selective NSAID, diclofenac, has been associated with cardiac reprogramming in postnatal and adult fibroblasts, downregulation of inflammatory mediators and fibroblasts genes and upregulation of cardiac genes. Declofenac also enhanced cardiac reprogramming through increased generation of cardiomyocytes, above that of known promoters of cardiac reprogramming such as TGFβ and Wnt inhibitors (Muraoka et al., 2019). These studies suggest that COX-2 selective NSAIDs may contribute to cardiac tissue repair although more validating work is needed.

### 2.4 Cytokine therapy

Cytokines may have a prominent role in mediating adverse cardiac remodelling. Cytokine expression profiles vary throughout the stages of cardiac remodelling from the initiation of an immune response through to the resolution phase following an ischemic insult. Several innate immune pathways are activated in the infarcted myocardium with inflammatory cytokines such as TGF-β, IL-1β, IL-6, IL-11, TNF-α and VEGF consistently detected at sites of damage (Li, Wang, Jia, & Du, 2014), with dysregulation of cytokine activity being associated with progression of HF. Whilst integral to promoting healing and mediating cardiovascular events their expression and activity must be tightly regulated to maintain homeostasis. In this area, research has progressed from animal studies to clinical trials with particular focus on IL-1, IL-6, IL-11 and TNF.

#### 2.4.1 IL-1

Interleukin (IL)-1 is activated in response to cardiac damage. IL-1α is released by necrotic cardiomyocytes and initiates the early inflammatory response to cardiac damage through fibroblast activation (Lugrin et al., 2015). IL-1 signalling is also critical during the post-infarction inflammatory stage. Subsequent to IL-1α release, activation of IL-1β activates pro-inflammatory signalling in leukocytes and fibroblasts (Bujak et al., 2008). The CANTOS trial evaluated canakinumab, a monoclonal antibody targeting IL-1β, in MI patients and elevated high-sensitivity C-reactive protein (CRP). The use of canakinumab (150 mg) prevented long-term adverse cardiac events over 3.7 years compared to control group. However, the trial also showed more fatal infections and sepsis in the treatment group despite patients with chronic or recurrent infection being excluded (https://www.acc.org/latest-in-cardiology/clinical-trials/2017/08/26/08/35/cantos). Several small studies have shown controversial findings associated with the use of anakinra, a recombinant human IL-1 receptor antagonist which competitively inhibits binding of IL-1α and IL-1β. In 10 patients with ST-segment elevation myocardial infarction (STEMI) treated with anakinra 100 mg/day for two weeks, reduced adverse LV remodelling was observed after 14 weeks (Abbate et al., 2010). However, in another study where 30 STEMI patients received the same dose for two weeks there was no effect on LV function and remodelling versus controls (Abbate et al., 2013). Yet, a meta-analysis of two studies highlighted a higher incidence of HF in the placebo group (Abbate et al., 2013), while an RCT in 182 non-STEMI patients receiving the same dose of anakinra 100 mg/day versus placebo over 2 weeks, a higher rate of major adverse cardiac events was observed in the treatment group at 1-year follow-up (Morton et al., 2015). A membrane associated co-receptor for IL-1RI called TILRR (toll-like and IL-1 receptor regulator) has been indicated as a potential therapeutic target given its increased expression in disease states such as MI, monocyte activation, carotid ligation as well as in apolipoprotein-E and low-density lipoprotein receptor mice on a high-fat diet compared to controls. In TILRR knockout mice IL1RI levels were reduced and proinflammatory regulators were less expressed (Smith et al., 2017). Hence, targeting TILRR, and thereby downstream signalling cascades, may trigger a more specific intermediate modulatory effect on IL-1 mediated responses opposed to the traditional all or nothing effect (Mac Gabhann, 2017).

#### 2.4.2 IL-6

Interleukin-6 (IL-6) binds to the membrane IL-6 receptor (IL-6R) which induces homodimerization of the receptor subunit glycoprotein 130 (gp130), thus activating a functional receptor complex of IL-6, IL-6R, and gp130 (Mihara, Hashizume, Yoshida, Suzuki, & Shiina, 2012). IL-6 has been implicated in coronary arterial disease and associated with increased myocardial injury and mortality in acute coronary syndromes (The Interleukin-6 Receptor Mendelian Randomisation Analysis (IL6R MR) Consortium*, 2012; Zamani et al., 2013). Hence, its suppression has been regarded as potentially beneficial. In a study using a mice MI model of coronary ligation, animals received either intraperitoneal IL-6 receptor antibody, MR16–1, at 500 μg/body or IgG for control. The MR16–1-treated mice showed reduced LV dilatation, improved LV function at 7 and 28 days following MI, with higher survival rate (80.6 vs. 59.5%, *P* < 0.05), and with less cardiomyocyte hypertrophy and fibrosis/scarring vs controls. Hence, this approach could represent an effective immune-modulating strategy following MI (Kobara et al., 2010). In the only human RCT available testing this strategy utilising the humanized anti-IL-6 receptor antibody drug, tocilizumab, MI patients were randomized at a median of 2 days after onset of symptoms to either placebo (*n* = 59) or tocilizumab (*n* = 58) as a single dose prior to coronary angiography. The use of tocilizumab was associated with lower levels of CRP and troponin levels and reduced inflammatory response with reduced levels of leukocyte and neutrophil concentration (Kleveland et al., 2016). A recent MI study in mice found that upregulation of gp130, the subunit protein of the activated IL-6 receptor complex, leads to increased cardiomyocyte proliferation at 7 days post MI, improved function and reduced infarct size at 1 month versus controls. The authors interpreted these findings as signs of cardiac regeneration, speculating that macrophage recruitment was essential in this context through the secretion of oncostatin M, a co-receptor for gp130. Hence, harnessing the gp130 subunit of IL-6 receptor complex to activate alternate pathways using gene therapy with adenovirus-associated virus encoding constitutively activated gp130 to promote heart regeneration may prove a potential therapeutic target in the clinical setting (Liu et al., 2020).

#### 2.4.3 IL-11

IL-11 is a member of the IL-6 cytokine family. Upon binding to its receptor, IL11RA1, it forms a receptor complex along with the subunit gp130. However, whereas IL-6/gp130 signalling occurs via the canonical Jak-STAT pathway, the IL-11 receptor complex predominantly activates the non-canonical pathway via ERK in mouse and human cardiac fibroblasts (Heinrich et al., 2003; Schafer et al., 2017). In the heart, IL-11 is highly specific to cardiac myofibroblasts, while IL-11 levels are virtually undetected in healthy tissue and cells (Schafer et al., 2017). mRNA IL-11 levels were markedly upregulated (>50 fold) in mice hearts following MI and remained elevated over 14 days (Obana et al., 2010). Plasma IL-11 levels are also elevated in CHF patients (*n* = 240) compared to patients without heart disease (*n* = 80), with IL-11 levels being correlated with adverse cardiac events, death and rehospitalisation (Ye et al., 2019). It has been postulated that neutralising anti-IL11 antibody (X203) might reduce cardiac fibrosis. In a mouse model of transverse aortic constriction, animals received either 20 mg/kg of X203 or isotype-control antibody twice-weekly for 2 weeks, 24 h after injury. In a further mouse model of chronic angiotensin II infusion (AngII), animals received 20 mg/kg of X203 or isotype-control antibody twice-weekly for 4 weeks, beginning 24 h after osmotic pump insertion. In both studies, X203 reduced pro-fibrotic gene expression and myocardial fibrosis (total collagen quantification) (Corden et al., 2021). Another study in mice MI using intravenous human recombinant IL-11 (rhIL-11) (3, 5 or 8μg/kg) every 24 h for 5 days versus controls revealed a reduction in cardiac fibrosis and apoptosis as well as increased capillary density at the infarcted site 14 days after MI (Obana et al., 2010). Subsequently, recombinant mouse IL-11 (rmIL-11) was administered in the same mice model daily for 6 days, resulting in conflicting results with stimulation of epicardial fibroblasts and a reduction in LVEF compared to controls (Schafer et al., 2017). This difference could be due to mice and humans sharing only 88% amino acid sequence homology for IL-11 (Keith, 2003), leading to functional differences between the species-specific protein tertiary structures. Evidence suggests that IL-11 is upregulated in response to TGFβ stimulation of cardiac fibroblasts, with neutralising of anti-IL-11 antibodies attenuating the pro-fibrotic effects of TGFβ1 suggesting that IL-11 has a pathogenic effect on the heart (Schafer et al., 2017). Hence, attempts have been made at targeting IL-11 as a therapeutic strategy to prevent LV adverse remodelling following MI. In a feasibility/safety pilot study 4 MI patients received the rhIL-11 clinical drug, oprelvekin, as a cardioprotective therapy with no adverse effects observed (Nakagawa et al., 2016). However, no further evidence is available on this potential therapeutic approach. Overall, the available data on IL-11 is conflicting, with a fundamental gap in research on cardiac repair following MI. Hence, more work is required, possibly in advanced large animal models, probing to determine if IL-11 exerts a pleiotropic effect following MI; perhaps being cardioprotective during the acute phase but leading to aberrant fibrotic signalling during the chronic phase, or whether the observed conflicting results are due to differences in study design, type of model used, or type/dose of IL-11 therapy tested.

#### 2.4.4 TNFα

TNF inhibitors include approved drugs to mediate the inflammatory response in patients with rheumatoid arthritis (RA), plaque psoriasis, ulcerative colitis and Crohn’s disease (Li, Perez-Chada, & Merola, 2019). Preclinical studies in small rodent MI have also shown a reduction in MI size following treatments with TNF-inhibitory agents (Liu et al., 2011; Sugano et al., 2004; Toufektsian et al., 2008). In addition, TNF inhibition improved LV pressure, diastolic function, wall thickening, and reduced leukocyte infiltration compared to controls in rat (Berry et al., 2004), while in a porcine model of MI leading to ventricular fibrillation (VF), animals treated with TNF blockade drug infliximab (5 mg/kg, *n* = 16) versus controls (n = 16) showed improvement in survival and early hemodynamic function (Niemann, Youngquist, Shah, Thomas, & Rosborough, 2013). This promising preclinical work has prompted 3 clinical trials using TNF-a antagonists in cardiac patients, although with controversial results. The ATTACH trial in 150 CHF patients suggested no benefits at 6-week associated with the use of infliximab, and possible detrimental effects with worsening of HF when high dose of infliximab (10 mg/kg) was used (Chung et al., 2003). Two additional trials focused more specifically on patients with chronic HF defined as LVEF ≤0.30. In the RECOVER trial, patients received either placebo (*n* = 373) or etanercept (25 mg weekly, *n* = 375) or etanercept (25 mg twice per week; n = 375). In the RENAISSANCE trial, patients received either placebo (*n* = 309), or etanercept (25 mg twice per week; *n* = 308), or etanercept (25 mg three times per week; n = 308). However, both trials were prematurely terminated due to lack of predefined clinical outcome benefits, for patients at the time the trial was terminated (Mann et al., 2004). Yet, a previous smaller trial in 47 patients showed improved LV function in the etanercept group (5 mg/m^2^ or 12 mg/m^2^ subcutaneously twice weekly for 3 month) compared to placebo control (Bozkurt et al., 2001). It is possible that these controversial results might be due to methodological differences across these trials. Additionally, the detrimental effect associated with the use of high dose treatment with TNF-antagonists observed in one of the 3 larger human trials cannot be ignored, as it suggests that obliteration of cellular signalling leads to adverse effects when measured by the real clinical setting. Of note, human trials have only focused on HF patients, hence there is no human data on the effects of TNF in modulating cardiac tissue repair following acute MI, given the beneficial effects observed with TNF antagonists in other clinical conditions such acute rheumatoid arthritis (Hartman, Groot, Leach, Karper, & van der Harst, 2018).

### 2.5 Chemokine therapy

Chemokines are a family of chemotactic cytokines which play a critical role in homeostasis and disease. They are classified into 4 subfamilies (CC, CXC, CX3C and XC) and initiate signalling by interacting with G-protein-coupled seven-transmembrane chemokine receptors (Griffith, Sokol, & Luster, 2014). The upregulation of chemokines, in particular CC and CXC, is a hallmark of post infarct inflammatory response which leads to leukocyte trafficking. CXC chemokines such as CXCL8/IL-8, are secreted in the infarct (Ivey, Williams, Collins, Jose, & Williams, 1995), and primarily stimulate recruitment of neutrophils, whereas CC chemokines, such as monocyte chemoattractant protein-(MCP)-1/CCL2 and CCL7, mediate recruitment of pro-inflammatory monocytes. Mechanisms influencing increased expression of inflammatory chemokines have been associated with the release of IL-1β as DAMPs, the activated inflammasome, oxidative stress and mechanical stress overload in the myocardium (Feng et al., 2015; Stevenson et al., 2019; Suetomi, Miyamoto, & Brown, 2019; Wang et al., 2018). Approaches targeting chemokines involved in recruitment of pro-inflammatory leukocytes have shown promising experimental results.

#### 2.5.1 CCL2

The CC chemokine MCP-1/CCL2 is rapidly upregulated in the infarcted myocardium and it is suggested that in acute coronary syndrome elevated baseline levels of MCP-1/CCL2 plasma levels are associated with an increased risk of MI or death (de Lemos et al., 2003). A mouse MI model with gene therapy deletion of MCP-1/CCL2 improved the survival rate at 4 weeks (61% versus 87%, *P* < 0.05), reduced contractile dysfunction, interstitial fibrosis, recruitment of macrophages, and myocardial gene expression inflammatory cytokines, but did not impact on infarct size vs controls (Hayashidani et al., 2003). In a mouse model of induced cardiomyopathy through repetitive ischemia/reperfusion injury MCP-1/CCL2 gene therapy deletion was associated with reduced interstitial fibrosis, macrophage infiltration, and improved LV function versus controls. Of note, in the same study similar results were obtained using MCP-1 neutralization antibody in WT mice (Frangogiannis et al., 2007). In an MI model using atherosclerotic prone (ApoE−/−) mice, the use of monocyte-directed RNAi targeting of CCR2, the main receptor for CCL2, resulted in reduced LV adverse remodelling, improved LVEF and reduction of key inflammatory genes such as MCP-1, IL1B, IL-6, TNF, with upregulation of IL-10 gene compared with controls (Majmudar et al., 2013).

#### 2.5.2 CCL5

A specifically designed compound, MKEY, which blocks the CCL5-CXCR4 heterodimerization interaction was investigated in a mouse MI model. Animals received intravenously MKEY or scrambled control (sMKEY) for 7 days after MI. The use of MKEY reduced infarct size, improved heart function an reduced tissue leukocyte recruitment (Vajen et al., 2018). Whilst additional research on CCL5 and cardiac regeneration is lacking, an interesting study has recently associated CCL5 as a novel chemokine with optic nerve regeneration while inhibition of one of CCL5's co-receptors, CCR5, reduced optic nerve regeneration by 72% (Xie, Yin, & Benowitz, 2021). Hence, CCL5 may represent a potential link in regulating cardiac tissue repair via the integrated nervous system and immune system processes referred to as the “super system”.

#### 2.5.3 CCL25

CCL25 promotes proliferation and chemotaxis of inflammatory cells that express its specific receptor, CCR9 (Igaki et al., 2018). CCR9 protein levels are increased in failing human hearts and hypertrophic murine mice (Xu, Mei, Liu, Sun, & Zheng, 2016). In a mouse MI model, CCL25 and CCR9 were up-regulated following MI, while CCR9 deficient mice had improved survival rate, LV function, and reduced infarct size compared to CCR9 positive mice. In addition, CCR9 deficient MI mouse hearts expressed more Bcl-2 and less Bax and cleaved caspase 3, indicating attenuation of apoptosis in cardiomyocytes. Furthermore, pro-inflammatory cytokines mRNA levels (IL-6, IL-1β, and TNF-α) were reduced (Huang et al., 2016). In a mouse model of pressure overload–induced cardiac hypertrophy via aortic banding, CCR9-deficient mice showed reduced LV diameters and interstitial fibrosis versus controls (Xu et al., 2016). Inhibition of CCR9 was also found to normalise ion currents, return calcium levels to homeostasis and maintain action potential duration following MI, indicating that CCR9 is a promising therapeutic target to treat and limit MI-induced arrhythmia (Huang et al., 2021). This preliminary data in mice warrants further validation in a large animal model.

#### 2.5.4 CXCL12

CXCL12 has been studied to a large extent in cardiac tissue repair with suggestions that it may have pro-angiogenic effects and promote activation of pro-survival pathways in cardiomyocytes (Bianchi & Mezzapelle, 2020). However, modulation of CXCL12 signalling through one of the main co-receptors, CXCR4, in MI models has led to conflicting results possibly due to the pleiotrophic effects of CXCR4 in many cell types (Frangogiannis, 2011). A study in mice MI has suggested that CXCR4 signalling does not play a crucial role in cardiomyocytes protection (Agarwal et al., 2010). CXCL12 modulation has been tried also through another of its main receptors, CXCR7 (Koch & Engele, 2020), and has recently been associated with neoangiogenesis and reduced apoptosis in a mouse MI model (Zhang et al., 2020). This finding supports the hypothesis that the protective mechanism of CXCL12 is exerted through activation of CXCR7 signalling within the infarcted myocardium. Accordingly, studies using synthetic analogs of CXCL12 have produced promising results. In a MI rat model, CXCL12 injected within a biomimetic hydrogel reduced infarct size and improved angiogenesis (Song et al., 2014). In a sheep MI model, administration of CXCL12 analog preserved LV function (Macarthur Jr. et al., 2014). The STOP-HF trial in ischemic HF patients suggested the safety of a single dose of plasmid stromal cell-derived factor-1 (pSDF-1/CXCL12) injected via endomyocardial route. However, the trial failed to demonstrate efficacy based on the proposed composite score at 4 months. Yet, a pre-specified sub-analysis suggested that the proposed treatment might reduce adverse LV remodelling and improve LVEF (Chung et al., 2015). Although these studies support CXCL12 as a potential therapeutic approach, more validation is needed. In addition, the pleiotropic and cell-specific actions of CXCL12 may require a dual-targeted approach to inhibit co-receptor activation, which in turn may exert pro-inflammatory responses to CXCL12.

#### 2.5.5 Concluding remarks

This review has focused only on preclinical and clinical in-vivo studies testing novel therapeutic approaches aimed at targeting neuro-immune systems to trigger cardiac tissue repair following MI. The review has identified several treatments associated with either beneficial or detrimental effects on myocardial healing post MI. This body of evidence confirm the basic concept that it is possible to influence cardiac tissue repair/function after MI by targeting the neuro-immune systems. By transition, these findings also confirm the presence of an overarching synergic response or molecular crosstalk occurring across the neuro-immune systems in response to MI, which appear to be bidirectional. The effects of these overarching activities appear to impact cardiac tissue repair in different ways in the immediately neonatal age as opposed to the adult phase as well as in the acute phase as opposed to the chronic phases of myocardial healing following MI. The review of these therapeutic approaches has highlighted those appearing to be safe as opposed to those appearing to be detrimental. Within those approaches appearing to be safe the review has highlighted conflicting results related to efficacy possibly due to methodological differences, and those treatments that may benefit from additional validation given their promising preliminary results.

The review has also suggested that preclinical research on modulating cardiac neuronal activity to trigger cardiac tissue repair post-acute MI has been meaningful. Therefore, a number of feasibility/preliminary clinical trials have been carried out in patients, although these have focused mostly on chronic MI patients with heart failure, with suggestion of improved cardiac function and quality of life (Buckley, Shivkumar, & Ardell, 2015). Underpinning mechanisms of nerve–mediated regulation and its immuno-regulatory effect on cardiac tissue repair are not yet clear. Further work is required to determine how the neuro-immune systems are integrated, and how these can be best modulated to promote more effective myocardial repair in the adult heart.

## Figures and Tables

**Table 1:** In-vivo studies using molecules to trigger cardiac tissue repair.

Cell Type	Molecule	Method	Model	MI Type	Outcome	Authors
Cardiomyocytes	Glucocorticoid Receptor	Knock Out	Mouse	AMI	↑ Cardiomyocyte proliferation ↓ Scar Size ↓ Mortality	Pianca et al., 2022
Cardiomyocytes	Mineralocorticoid Receptor	Knock Out	Mouse	AMI	↓ Cardiac Remodelling ↓ Apoptosis	Fraccarollo et al., 2011
Cardiomyocytes	Glucocorticoid Receptor	Knock Out	Mouse	No MI	↓ LVEF ↑ LV Mass ↑ Hypertrophy ↑ Premature death from HF	Oakley et al., 2013 Oakley et al.,2019
Cardiomyocytes	Glucocorticoid Receptor	Knock Out	Mouse	No MI	Normal lifespan Normal cardiac morphology Normal cardiac function	Oakley et al., 2019
Cardiomyocytes	Glucocorticoid Receptor and Mineralocorticoid Receptor	Knock Out	Mouse	No MI	Resistent to LV dysfunction Resistent to adverse remodelling Normal lifespan	Oakley et al., 2019
Global	11β-HSD1	Knock Out	Mouse	CMI	↑ LVEF ↓ Hypertrophy ↓ Scar Size	White et al., 2016
Global	11β-HSD1	Inhibitor	Mouse	Non perfused cardiac remodelling	Reversed LV hypertrophy Reversed LV dysfunction	Gordon et al., 2014
Global	Interleukin-6	Antibody	Mouse	MI	↑ LVEF ↓ Fibrosis ↓ Hypertrophy ↑ Survival	Kobara et al., 2010
Cardiomyocytes	gp130	Cre activated	Mouse	MI	↑ LV Function ↓ Scar Size	Li et al., 2020
Global	Interleukin-11	Human recombinant protein	Mouse	MI	↓ Fibrosis ↓ Apoptosis	Obana et al., 2010
Global	Interleukin-11	Mouse recombinant protein	Mouse	MI	↓ LVEF ↑ Activated Fibroblasts	Schafer et al., 2017
Global	Interleukin-11	Neutralising antibody	Mouse	Pressure overload	↓ Myocardial Fibrosis	Corden et al., 2021
Global	CCL2 (MCP-1)	N-terminal deletion mutant of MCP-1. Plasmid administered	Mouse	MI	↑ LV Function ↑ Improve Survival ↓ Interstitial Fibrosis No difference in scar size	Hayashidani et al., 2003
Monocytes	CCR2	RNA Interference	Mouse	MI	↑ LV Function ↓ LV Remodelling	Majmudar et al., 2013
Global	CCL5/CXCR4	Interaction Inhibitor	Mouse	MI	↓ Scar Size ↓ Leukocyte recruitment	Vajen et al., 2018
Global	CCR9	Knockout	Mouse	MI	↓ Scar Size ↓ Apoptosis ↑ LV Function ↑ Survival	Huang et al., 2016
Global	CXCR7	CXCR4 antagonist	Mouse	MI	↓ Apoptosis ↑ Angiogenesis	Zhang et al., 2020
Infarct border zone territory	CXCL12	Hydrogel composite injected into the epicardium	Rat	MI	↓ Scar size ↑Angiogenesis	Song et al., 2014
Infarct border zone territory	CXCL12	Border zone Injection	Sheep	MI	↓ Scar size ↑ Angiogenesis	Macarthur Jr. et al., 2014

AMI: Acute Myocardial Infarction; LVEF: Left Ventricular Ejection Fraction; LV: Left Ventricle; HF: Heart Failure; CMI: Chronic Myocardial Infarction.

**Table 2:** In-vivo preclinical studies using drugs to trigger cardiac tissue repair.

Molecule	Medication	Model	MI Type	Outcome	Authors
COX2	Parecoxib	Rat Mouse	MI	↓ LV Remodelling ↓ Cell death	Abbate et al., 2006 Straino et al., 2007
COX2	Parecoxib	Mouse	Non Perfused MI	↓ LV Remodelling ↓ Cell death	Salloum et al., 2009
COX2	Parecoxib	Mouse	Perfused MI	No difference in mortality of cell death	Salloum et al., 2009
COX2	Celecoxib	Mouse	Cryoinjury	↓ Hypertrophy ↓ Fibrosis ↑ Cardiac Function	Zhao et al., 2021
COX2	Celecoxib	Pig	MI	↑ Mortality ↑ LV Redmodelling ↓ Systolic Function	Timmers et al., 2007
TNFα	Etanercept	Rat	MI	↑ LV Pressure	Berry et al., 2004
TNFα	Infliximab	Pig	MI	↑ Survival ↑ LV Function	Niemann et al., 2013

MI: Myocardial Infarction; LV: Left Ventricle.

## Abbreviations

11β-HSD1: 11βeta-hydroxysteroid dehydrogenase type 1
AMI: Acute myocardial infarction
AngII: Angiotensin II
ApoE−/−: Apolipoprotein-E knock out
*AR receptor*: *Androgen receptor*
AT-1 receptor: Angiotensin II *receptor* type 1
β-blocker: Beta blocker
β1-AR: Beta-1 adrenergic receptor
Bax: Bcl-2-associated X protein
Bcl-2: B-cell lymphoma 2
cAMP: Cyclic adenosine monophosphate
CCL2: Chemokine (C-C motif) ligand 2
CCL5: Chemokine (C-C motif) ligand 5
CCL7: Chemokine (C-C motif) ligand 7
CCL25: Chemokine (C-C motif) ligand 25
CCR2: Chemokine receptor type 2
CCR5: Chemokine receptor type 5
CCR9: Chemokine receptor type 9
cGR-KO: Cardiomyocyte-specific knockout of the glucocorticoid receptor
cGR-cMR-KO: Cardiomyocyte-specific knockout of both glucocorticoid receptor and mineralocorticoid receptor
CHF: Chronic heart failure
CMI: Chronic myocardial infarction
cMR-KO: Cardiomyocyte-specific knockout of the mineralocorticoid receptor
COX: Cyclooxygenase
CRP: C-reactive protein
CXCL8: C-X-C motif chemokine 8
CXCL12: C-X-C motif chemokine 12
CXCR4: C-X-C chemokine receptor type 4
CXCR7: C-X-C chemokine receptor type 7
DAMPs: Damage-associated molecular patterns
DCM: Dilated cardiomyopathy
GC: Glucocorticoid
gp130: Glycoprotein 130
GR: Glucocorticoid receptor
HF: Heart Failure
IFN-γ: Interferon Gamma
IL-1: Interleukin 1
IL-1α: Interleukin 1 alpha
IL-1β: Interleukin 1 beta
IL-1R: Interleukin 1 Receptor
IL-1RI: Interleukin 1 Receptor Type 1
IL-6: Interleukin 6
IL-6R: Interleukin 6 Receptor
IL-8: Interleukin 8
IL-10: Interleukin 10
IL-11: Interleukin 11
IL11RA1: Interleukin 11 Receptor subunit alpha-1
IVIg: Intravenous immunoglobulin
LV: Left ventricle
LVEF: Left ventricular ejection fraction
M2: Type 2 macrophage
MCP-1: Monocyte chemoattractant protein 1
MI: Myocardial infarction
MR: Mineralocorticoid receptor
Na-K-ATPase: Sodium-potassium-adenosine triphosphate-ase
NF-KB: Nuclear factor kappa B
NSAID: Nonsteroidal anti-inflammatory drug
NSTEMI: Non-ST-segment elevation myocardial infarction
pSDF-1: Plasmid stromal cell-derived factor-1
Pyr: Pyridostigmine
RCT: Randomized controlled trial
rhIL-11: Recombinant human Interleukin 11
rmIL-11: Recombinant mouse Interleukin 11
RNAi: Ribonucleic acid interference
STEMI: ST-segment elevation myocardial infarction
TGF-β: Transforming growth factor beta
TILRR: Toll-like and IL-1 receptor regulator
TNF: Tumor necrosis factor
TNF-α: Tumor necrosis factor alpha
Treg: Regulatory T-cell
VEGF: Vascular endothelial growth factor
VF: Ventricular fibrillation
Wnt: Wingless-related integration site
WT: Wild-type
